# Survival on antiretroviral treatment among adult HIV-infected patients in Nepal: a retrospective cohort study in far-western Region, 2006–2011

**DOI:** 10.1186/1471-2334-13-604

**Published:** 2013-12-26

**Authors:** Laxmi Bhatta, Elise Klouman, Keshab Deuba, Rachana Shrestha, Deepak Kumar Karki, Anna Mia Ekstrom, Luai Awad Ahmed

**Affiliations:** 1 Department of Community Medicine, Faculty of Health Sciences, University of Tromsø, Tromsø, Norway; 2 Department of Public Health Sciences, Karolinska Institutet, Stockholm, Sweden; 3 Centre for International Health, Faculty of Medicine and Dentistry, University of Bergen, Bergen, Norway; 4 National Centre for AIDS and STD Control, Kathmandu, Nepal; 5 Division of Global Health (IHCAR), Department of Public Health Sciences, Karolinska Institutet, Stockholm, Sweden; 6 Department of Health and Care Sciences, Faculty of Health Sciences, University of Tromsø, Tromsø, Norway

**Keywords:** HIV, Antiretroviral treatment (ART), Mortality, Determinants, Far-western region, Nepal, Asia

## Abstract

**Background:**

Though financial and policy level efforts are made to expand antiretroviral treatment (ART) service free of cost, survival outcome of ART program has not been systematically evaluated in Nepal. This study assesses the mortality rates and determinants among adult HIV-infected patients on ART in Far-western region of Nepal.

**Methods:**

This retrospective cohort study included 1024 (51.2% men) HIV-infected patients aged ≥15 years, who started ART between May 15^th^ 2006 and May 15^th^ 2011 in five ART sites in the Far-western region, Nepal. Follow-up time was calculated from the date of ART initiation to date of death or censoring (loss to follow-up, transferred out, or 15 November 2011). Mortality rates (per 100 person-years) were calculated. Kaplan-Meier and Cox-regression models were used to estimate survival and explore determinants of mortality.

**Results:**

The median follow-up time was 19.1 months. The crude mortality rate was 6.3 (95% confidence interval (CI) 5.3-7.6) but more than three-times higher in first 3 months after ART initiation (21.9 (95% CI 16.6- 28.8)). About 12% (83% men) of those newly initiated on ART died during follow-up. The independent determinants of mortality were male sex (hazard ratio (HR) 4.55, 95% CI 2.43-8.51), poor baseline performance scale (bedridden <50% of the day during the past month, HR 2.05, 95% CI 1.19-3.52; bedridden >50% of the day during the past month, HR 3.41, 95% CI 1.67-6.98 compared to normal activity), one standard deviation decrease in baseline bodyweight (HR 1.04, 95% CI 1.01-1.07), and poor WHO clinical stage (stage III, HR 2.96, 95% CI 1.31-6.69; stage IV, HR 3.28, 95% CI 1.30-8.29 compared to WHO clinical stage I or II).

**Conclusions:**

High mortality was observed within the first 3 months of ART initiation. Patients with poor baseline clinical characteristics had higher mortality, especially men. Earlier initiation of ART through expanded testing and counselling should be encouraged in HIV-infected patients.

## Background

It has been estimated that antiretroviral treatment (ART) has averted 300,000 HIV-related deaths in Asia [1]. Although it is well established that ART reduces mortality and prevent opportunistic infection among HIV patients in high-income countries and in generalized epidemics in Africa, mortality rates and mortality determinants among ART-recipients in countries with concentrated epidemics in the South Asian region have not been well-covered [2-6]. There is reason to believe that there are large regional variations in terms of treatment outcomes due to poor access to services in particular among most at risk of HIV-infection and minority populations with stigmatized risk behaviours [4,7,8].

Nepal is among the poorest countries in the world and health programs, especially HIV programs are heavily donor dependent [7]. Nepal has a concentrated HIV epidemic with a low estimated HIV prevalence of 0.30% among adults (15–49 years) in the general population but over 5-fold higher rates among populations at higher risk including male labour migrants (particularly to India where they often visit sex workers), men who have sex with men, female sex workers, people who inject drugs [7,9]. These population groups are considered important bridging populations for HIV transmission between high-risk and low-risk shares of the populations [7,10].

Since the introduction of free ART services in Nepal in 2004 with a primary aim to reduce mortality among HIV-infected patients, there have been improvements in service delivery and utilization. In addition to the free-of-cost highly affective antiretroviral therapy, HIV-infected patients are offered care package including clinical follow-up monitoring, TB screening and Isoniazid preventive therapy, and community and home-based care (CHBC), which includes primary care of patients by trained CHBC workers at homes and community settings aiming for positive living, reducing stigma and discrimination if any, and supporting for sanitation and hygiene, continuity and adherence to ART. Moreover, medical, nutritional, psychosocial, legal support is provided by CHBC, and in addition financial support is provided to newly enrolled patients by community care centers nearby ART sites [11,12]. However, the ART coverage of people eligible to treatment is still low despite a doubling from 10% in 2007 to 24% of those in need (having a CD4 count <350 cells/mm^3^) in 2011 [9,13]. No previous systematic review has been done in Nepal, but it is needed to better understand the most important determinants of mortality among patients initiated on ART in order to identify those at the highest risk of preventable death. This cohort study explores the mortality rates and determinants among all known ART patients in one of the five development regions of Nepal, the Far-western region, in a thorough retrospective assessment including the most recent available data since the start of ART in this area, i.e. from 2006 to 2011.

## Methods

### Study setting

The Far-western region (population 2.5 million [14]) is a remote and developmentally challenged region of Nepal [15]. There are seven ART sites in the Far-western region of Nepal. All the ART sites in the region are run by government hospitals except Bayalpata hospital in Achham district that is run by a non-governmental organization. Five ART sites (Seti Zonal Hospital and Tikapur Hospital, Kailali district; Mahakali Zonal Hospital, Kanchanpur district; Achham District Hospital, Achham district; Doti District Hospital, Doti district) in the region have treatment initiation capabilities, while two ART sites (Baitadi District Hospital, Baitadi district; Bayalpata Hospital, Achham district) only offer treatment follow-up for transferred-in HIV-positive patients who were tested and started the treatment at other ART sites within or outside the region. Patients were transferred to these two sites in order to ease their access to treatment near their place of residence. These two sites were ineligible for this study due to lack or incomplete baseline information and ART initiation date.

### Study population

**Figure 1:**
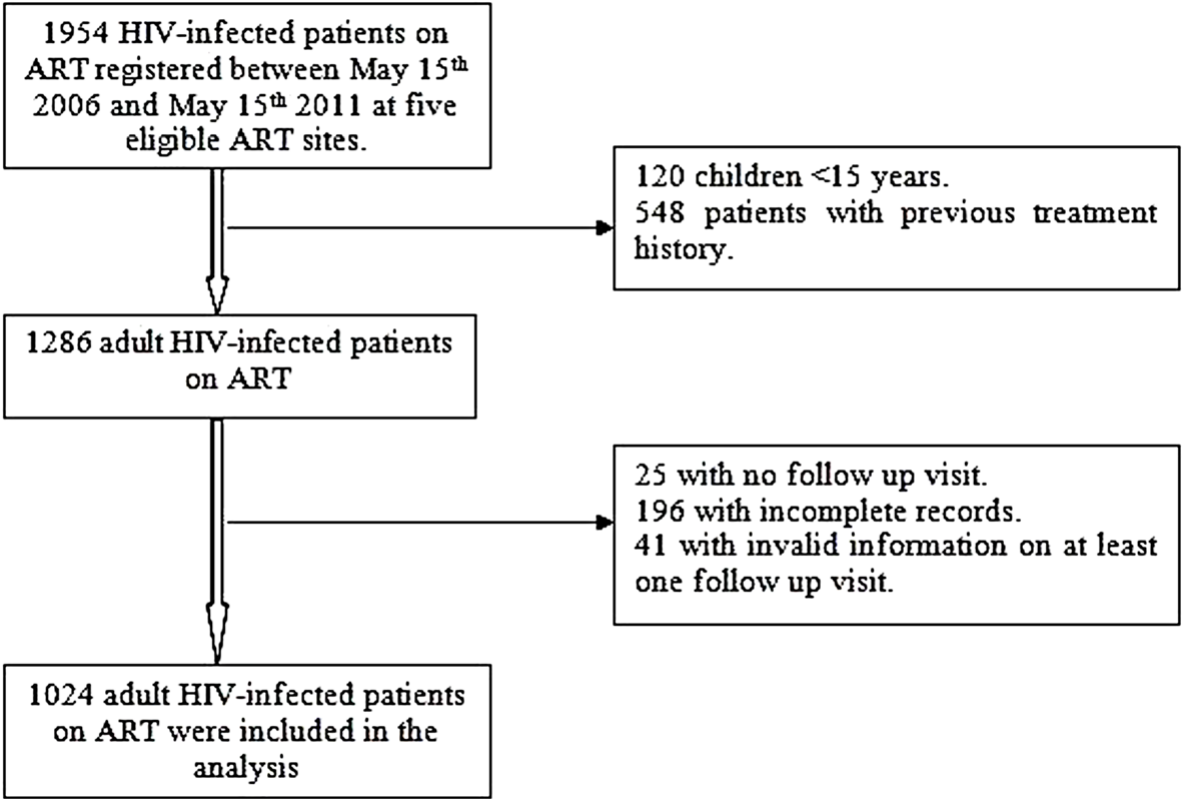
Profile of the study cohort.

The study population consisted of all adult HIV-infected patients (15 years of age and older) on ART, who started the treatment between May 15^th^ 2006 (first recipient recorded in ART site of Far-western region) and May 15^th^ 2011 at any of the five eligible ART sites in the Far-western region. All patients with history of previous treatment, and children aged 0–14 years -as defined by the National Centre for AIDS and STD control (NCASC), Ministry of Health and Population (MoHP), Nepal [9] were excluded from the study. The total eligible population was 1286 adult HIV-infected patients and they were followed-up with respect to death until 15 November 2011 (Figure 1).

### Data collection and study variables

Data were collected from standard medical record registers at ART sites adopted by the NCASC, Nepal. There were three available registers. The first register was the Pre-ART register where all confirmed HIV-positive clients who visit ART site were registered. Then, all the patients who started ART regimen were transferred to an ART register at the date of treatment initiation. The third register was patient’s follow-up form. For every patient, the first follow-up visit was scheduled two weeks after treatment initiation and then on a monthly basis, where medical records were updated for every patient during each follow-up visit. Data collection approval was obtained from all ART sites. Confidentiality and anonymity of data was maintained during data collection. Ethical approval was obtained from the Nepal Health Research Council (NHRC), Kathmandu, Nepal.

Patients’ baseline demographic and clinical characteristics data were collected. Baseline demographic characteristics included place of ART site, age (in years), sex, and education. Clinical characteristics at the time of ART initiation included performance scale, bodyweight (kg), World Health Organization (WHO) clinical stage, CD4 count (cells/mm^3^), ART regimen, drug toxicity/side effects, and active tuberculosis (TB) during treatment. Performance scale was categorized and recorded into three groups: normal activity, bedridden <50% of the day during the past month, bedridden >50% of the day during the past month [16]. The clinical stage of the HIV-infected patient was assessed and recorded using the WHO clinical stage guidelines [16] based on load of clinical symptoms and infections reported by the health professionals at ART sites. The WHO clinical stage was categorized into four groups (stage I, stage II, stage III, and stage IV), where stage IV is considered as the worst health condition [16]. The first line ART regimens in adults and adolescent are ZDV/3TC/NVP, ZDV/3TC/EFV, d4T/3TC/NVP, d4T/3TC/EFV, TDF/3TC/NVP, and TDF/3TC/EFV. In case of treatment failure, it is recommended to change to second line combination regimen. The second line regimens in adults and adolescent are TDF + 3TC + LVP/r and ZDF + 3TC + LVP/r [16].

The date of death was recorded for all HIV-infected patients, who died from all causes during the study period while on antiretroviral treatment. Patients missing their follow-up visits for more than 3 months were counted as loss to follow-up and the date of the last registered follow-up visit was recorded as date of loss to follow-up. ART using HIV-infected patient, who were transferred to another ART site, were recorded as transferred-out cases and their dates of transferred-out were also recorded.

### Data analysis

Adult HIV-infected patients who had no follow-up visits (n = 25) and, patients without date of ART initiation and date of occurrence of events (i.e. death, loss to follow up, and transferred out) (n = 196) were excluded from study. An additional 41 patients were excluded due to invalid follow-up information on at least one follow-up visit (for example, transferred out date earlier than first follow-up visit date). A total of 1024 adult HIV-infected patients were included in the present analysis (Figure 1).

Follow-up time (in months) was calculated from the date of ART initiation to the date of death or censoring (loss to follow-up, transferred out, or 15 November 2011). Kaplan-Meier (KM) models were used to estimate survival probability after ART initiation. All the baseline demographic and clinical characteristics were used as independent variables in the analysis. Proportional hazard assumption was assessed and all the independent variables satisfied the assumption. The Cox-proportional hazard model was used to assess the relationship between the independent variables and mortality. The univariate Cox-regression analysis was used to estimate the unadjusted Hazard Ratios (HRs), and the stepwise (backward LR) multivariate Cox-regression analysis (including all independent variables) was performed to estimate the adjusted hazard ratios. The probability for the stepwise regression was 0.05 for entry of the variables and 0.10 for removal of the variables from model. All the tests were two-sided and the criterion for statistical significance was set at *p* < 0.05. A worst-case-scenario analysis was performed assuming all lost to follow-up patients were dead immediately after the last date of contact. Data were analysed using the statistical software STATA version 12.0 and SPSS version 16.0.

## Results

Among the 1024 adult HIV-infected patients, 14 (1.4%) were lost to follow-up, 198 (19.3%) were transferred-out to other ART site within Far-western region or outside the region, 120 (11.7%) died, and 692 (67.6%) were still alive by the 15 November 2011. Among 120 patients who died, 51 (42.5%) died within 3 months after start of treatment. There was inadequate information regarding the causes of deaths among adult HIV-infected patients on ART.

**Figure 2:**
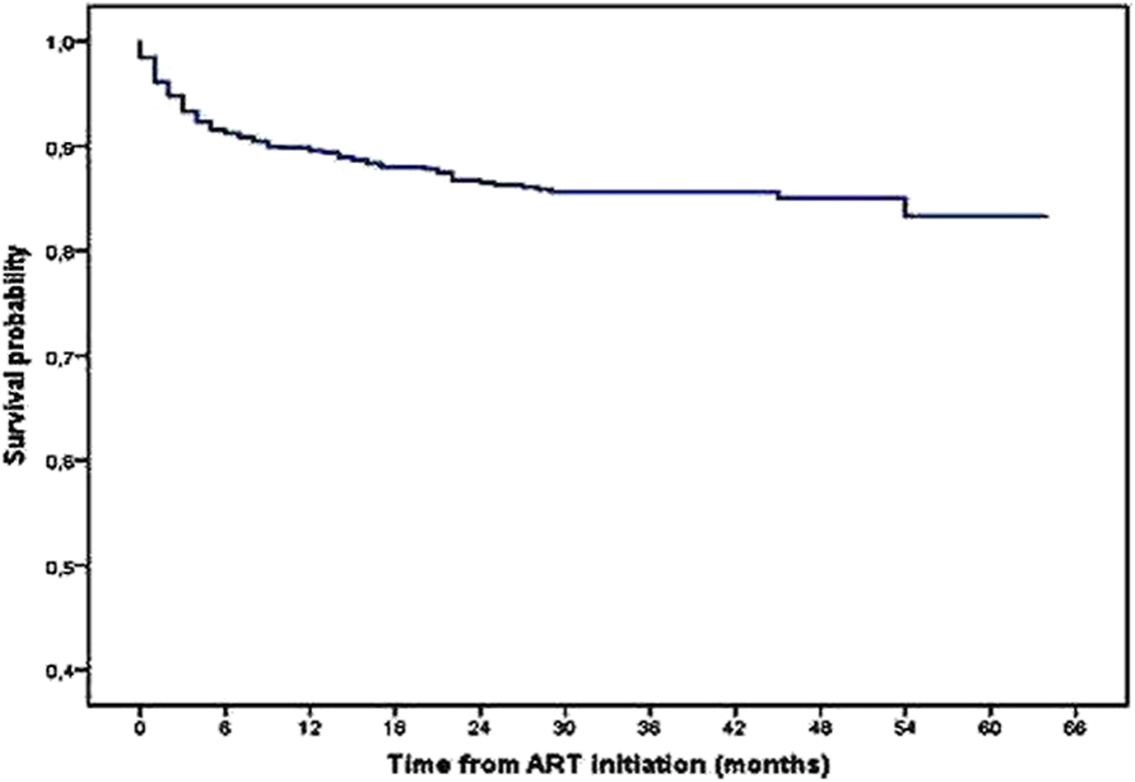
Kaplan-Meier survival curve of 1024 adult HIV-infected patients on ART.

The overall median follow-up time of all the patients was 19.1 months or 1.6 years (Interquartile range 0.6 – 2.9 years). The median follow-up time of the deceased (3.6 months) and lost to follow-up patients (13.2 months) was lower than median follow-up time of survivors (29.5 months). The overall survival probability among adult HIV-infected patients declined over follow-up time (Figure 2). The survival probability of patients at 3 month, 6 month, 1 year, 2 year, and at 5 year was 94.7%, 91.4%, 89.7%, 86.5% and 82.9% respectively.

The study cohort contributed a total of 1895 person-years of follow-up. The total mortality rate was 6.3 per 100 person-years at risk. The mortality rate was highest in the first three months after ART initiation (21.9 per 100 person-years (95% CI 16.6-28.8)) (Table 1).

**Table 1:** Mortality rates (per 100 person-years) of HIV-infected patients over different follow-up time intervals

**Follow-up time intervals**	**Mortality rate per 100 person-years at risk (95% CI)**
	(**N** = **1024**)
**0** – **3 months**	21.9 (16.6 – 28.8)
**0** – **6 months**	18.2 (14.6 – 22.6)
**0** – **1 year**	11.9 (9.7 – 14.5)
**0** – **2 years**	8.5 (7.1 – 10.3)
**0** – **5 years**	6.3 (5.3 – 7.6)
**Over the study period**	6.3 (5.3 – 7.6)

N = total number of patients.

The baseline demographic and clinical characteristics of adult HIV-infected patients on ART are summarized in Table 2. The median age of the patients at start of ART was 35 years (Interquartile range 30–42 years), and male patients constitute 51.2% of the total sample size. About 1 out of 11 patients were bedridden for >50% of the day during the past month. The median baseline bodyweight was 45 kg (Interquartile range 40–50). The median baseline CD4 count was 149 cells/mm^3^ (Interquartile range was 80–210). About 17% of patients were in WHO clinical stage IV, where one out of four died. The proportions of deaths varied between the ART sites (*p*-value for χ^2^ test was 0.001).

**Table 2:** Baseline demographic and clinical characteristics of 1024 patients on ART

**Characteristics**	**Number of patients (%)**^**a**^	**Number of deaths (%)**^**b**^
**Sex**		
Female	500 (48.8%)	21 (4.2%)
Male	524 (51.2%)	99 (18.9%)
**Age (years)**		
15 – 32 years	338 (33.0%)	27 (8.0%)
33 – 40 years	401 (39.2%)	52 (13.0%)
>40 years	285 (27.8%)	41 (14.4%)
**Education**		
Illiterate	650 (73.5%)	69 (10.6%)
Literate	234 (26.5%)	35 (15.0%)
*Missing data*	*140* (*13.6%*)	
**Baseline performance scale**		
Normal	732 (73.1%)	45 (6.1%)
Bedridden <50%	182 (18.2%)	44 (24.2%)
Bedridden >50%	87 (8.7%)	28 (32.2%)
*Missing data*	*23* (*2.2%*)	
**Baseline bodyweight (kg)**		
< 45 Kg	484 (49.1%)	58 (12.0%)
45 – 60 Kg	479 (48.6%)	49 (10.2%)
>60 Kg	23 (2.3%)	2 (8.7%)
*Missing data*	*38* (*3.7%*)	
**Baseline WHO clinical stage**		
Stage I or II	320 (34.2%)	10 (3.1%)
Stage III	455 (48.6%)	63 (13.8%)
Stage IV	161 (17.2%)	41 (25.5%)
*Missing data*	*88* (*8.6%*)	
**Baseline CD4 count (cells/mm**^**3**^**)**		
≤ 50 cells/mm^3^	127 (13.6%)	19 (15.0%)
51- 200 cells/mm^3^	533 (57.1%)	69 (13.0%)
>200 cells/mm^3^	274 (29.3%)	14 (5.1%)
*Missing data*	*90* (*8.8%*)	
**Drug toxicity/side effects**		
No	788 (80.9%)	83 (10.5%)
Yes	186 (19.1%)	31 (16.7%)
*Missing data*	*50* (*4.9%*)	
**Active TB during treatment**		
No	856 (83.6%)	89 (10.4%)
Yes	168 (16.4%)	31 (18.5%)
**Baseline ART regimen**^#^		
d4T/3TC/NVP	151 (14.8%)	26 (17.2%)
d4T/3TC/EFV	44 (4.3%)	7 (16.0%)
ZDV/3TC/NVP	702 (69.0%)	64 (9.1%)
ZDV/3TC/EFV	118 (11.6%)	20 (17.0%)
TDF/3TC/EFV	3 (0.3%)	0
*Missing data*	*6* (*0.6%*)	
**Baseline ART regimen change**		
No	760 (74.2%)	78 (10.3%)
Yes	264 (25.8%)	42 (16.0%)
**ART centre/sub centre**		
Seti Zonal hospital	590 (57.6%)	51 (8.6%)
Kanchanpur Zonal hospital	70 (6.8%)	13 (18.6%)
Achham District hospital	176 (17.2%)	24 (13.6%)
Doti district hospital	176 (17.2%)	32 (18.2%)
Tikapur hospital	12 (1.2%)	0

^a^column percentage (missing data was excluded);.

^b^row percentage;.

^#^all regimen were first line ART regimen.

Note: missing data refers to incomplete/missing recorded characteristics of patients.

Table 3 shows the hazard ratios from the univariate and multivariate analysis of the association between the possible determinants of mortality and risk of death. In univariate analysis, place of ART site, sex, age, baseline performance scale, bodyweight, WHO clinical stage, CD4 count, active TB during treatment, and ART regimen had statistically significant relation with the mortality in adult HIV-infected patients.

**Table 3:** **Hazard ratios** (**HR**) **of mortality in 1024 patients on ART**

**Determinants**	**Univariate analysis**		**Multivariate analysis**^***f***^	
	**HR (95% CI)**	***P*** **value**	**HR (95% CI)**	***P*** **value**
**Place of ART**^**a**^				
Kailali Distric^*b*^ (ref.)	1.00			
Kanchanpur District^*b*^	2.03 (1.10 – 3.73)	0.023		
Doti District^*c*^	1.93 (1.24 – 3.00)	0.004		
Achham District^*c*^	1.71 (1.05 – 2.78)	0.031		
**Sex**				
Female (ref.)	1.00		1.00	
Male	5.40 (3.37 – 8.65)	<0.001	4.55 (2.43 – 8.51)	<0.001
**Age groups** (**years**)				
15- 32 years (ref.)	1.00			
33- 40 years	1.66 (1.04 – 2.64)	0.033		
>40 years	1.93 (1.19 – 3.14)	0.008		
**Education**				
Illiterate (ref.)	1.00			
Literate	1.45 (0.96 – 2.17)	0.075		
**Performance scale**				
Normal (ref.)	1.00		1.00	
Bedridden <50%	4.54 (2.99 – 6.88)	<0.001	2.05 (1.19 – 3.52)	0.010
Bedridden >50%	5.53 (3.45 – 8.87)	<0.001	3.41 (1.67 – 6.98)	0.001
**Bodyweight** (**Kg**)^d^	1.02(1.01 – 1.05)^*g*^	0.048	1.04(1.01 – 1.07)^*g*^	0.010
**WHO clinical stage**				
Stage I or II (ref.)	1.00		1.00	
Stage III	4.39 (2.25- 8.55)	<0.001	2.96 (1.31- 6.69)	0.009
Stage IV	8.85 (4.43-17.66)	<0.001	3.28 (1.30- 8.29)	0.012
**CD4 count** (**cells**/**mm**^**3**^)^d^	0.99 (0.98- 0.99)	<0.001		
**Drug toxicity**/**side effects**				
No (ref.)	1.00			
Yes	1.45 (0.96- 2.19)	0.078		
**Active TB during treatment**				
No (ref.)	1.00			
Yes	1.73 (1.15- 2.61)	0.009		
**ART regimen**^**e**^				
d4T/3TC/NVP (ref.)	1.00			
d4T/3TC/EFV	0.90 (0.39- 2.07)	0.803		
ZDV/3TC/NVP	0.49 (0.31- 0.77)	0.002		
ZDV/3TC/EFV	0.97 (0.54- 1.73)	0.907		
**ART regimen change**				
No (ref.)	1.00			
Yes	1.37 (0.94- 2.00)	0.10		

^**a**^Kailali District (Seti Zonal Hospital and Tikapur Hospital), Kanchanpur District (Kanchanpur Zonal Hospital), Doti District (Doti District Hospital), and Achham District (Achham District Hospital). ^b^plain/terai district, ^c^hill district . ^d^Continuous variables. ^**e**^TDF/3TC/EFV regimen was not included. ^f^Cox proportional hazards model adjusted for all variables in the table. ^g^HR for 1 kg decrease in bodyweight.

*ref*.: reference group.

However, in the multivariate analysis, only sex, baseline performance scale, bodyweight, and WHO clinical stage were independent significant determinants of mortality. The risk of death increased 4.6-fold (95% CI: 2.43- 8.51) in male compared to female patients. Patients with baseline performance scale of bedridden <50% were two times more likely to die compared to patients with normal performance scale at start of treatment (HR 2.05; 95% CI: 1.19- 3.52). However, the risk of mortality increased 3.4 times when the patients had baseline performance scale of bedridden >50% compared to patients with normal baseline performance scale (HR 3.41; 95% CI: 1.67- 6.98). For each kilogram decrease in baseline bodyweight, the risk of mortality increased by 4% (HR 1.04; 95% CI: 1.01- 1.07). HIV-infected patients with WHO clinical stage III had 3-fold increased risk of death compared to patients with stage I or II (HR 2.96; 95% CI: 1.31- 6.69), and the risk of death among WHO clinical stage IV patients was even higher- compared to stage I or II patients (HR 3.28; 95% CI: 1.30- 8.29).

In stepwise (backward LR) multivariate analysis, categorizing baseline CD4 count (into three groups: ≤50 cells/mm^3^, 51–200 cells/mm^3^, and >200 cells/mm^3^) and place of ART site (into two groups: Hill district (Doti district and Achham district) and Plain/Terai district (Kailali district and Kanchanpur district)) showed no statistical significant relation with mortality among adult HIV-infected patients on ART (*data not shown*).

## Discussion

This 5-year retrospective cohort study showed high mortality within 3 months of ART initiation among HIV-infected patients in Nepal. The major independent determinants of mortality among adult HIV-infected patients on ART were male sex, poor baseline performance scale, low baseline bodyweight, and worse baseline WHO clinical stage.

In this study, 11.7% of the adult HIV-infected patients on ART died, similar with the overall deaths (11.8%) on ART program in Nepal by July 2011 [13]. The causes of deaths were not investigated in this study; however, it is well known that opportunistic infections (OIs) are the major causes of mortality among HIV-infected patients. In Nepal, the most prevalent OIs among HIV-infected patients are tuberculosis, candidiasis and cryptosporidiosis [17,18].

The overall mortality in this study is quite higher than in high-income [19], but in line with low-income countries [6,20]. This could indicate poor ART program initiatives and low coverage of the program in Nepal. Similar to other studies [5,6,21], the first 3 months mortality after ART initiation was the highest. The poor outcome in the first few months after ART initiation might be due to delayed diagnosis and/or treatment and explained by the fact that 70.7% patients had advanced disease (CD4 count ≤200 cells/mm^3^) and 65.7% patients had advanced clinical symptoms (WHO clinical stage III or IV) at the time of treatment initiation. Some factors such as stigma and discrimination related to HIV [10], limited availability and access to HIV testing and counselling (HTC) and ART services in most areas might have played role in delayed diagnosis and/or treatment. Moreover, lack of proper screening of OIs, limited availability of prophylaxis and diagnostic facility of OIs might also have increased the mortality [20,22]. Advanced clinical stage (stage IV) is strongly associated with high mortality during the first months of treatment [23].

The variations in the mortality rates across different countries indicate that effectiveness of ART to reduce the mortality and increase the survival among HIV-infected patients could depend on the adherence, quality of service, and characteristics of patients [4,24].

In Nepal, male HIV-infected patients reported through HTC by July 2012 were double than female [25]. However, the proportion of male ART receivers was only 10% more than female in Nepal [26]. This indicates that female patients tend to enrol more frequently in ART service than men, and they would have early initiation of treatment due to the linkage between the community-based prevention of mother-to-child transmission (CB-PMTCT) and treatment and care program. Taking PMTCT service through community level had dramatically increased its utilization by pregnant mothers and it might have effectively encouraged females to get to know their HIV status and start early treatment through awareness and counselling services [27,28].

This study showed that male adult HIV-infected patients had higher risk of mortality and this is similar to several [4,5,24], but not all previous studies [20,21], where the latter showed no significant relationship between sex and mortality. These differences between several studies might be due to differences in accessibility and utilization of health services offered and differences in study setting explaining cultural differences. Moreover, late reporting to treatment centre unless experiencing worst HIV related symptoms might be more prevalent among male patients [24]. In Mid-and Far-western region of Nepal, the HIV prevalence is about 5% among male labor migrants [29]. The high prevalence of HIV among male labor migrants, especially in the Far-western region of Nepal is associated both with poor knowledge about HIV, stigmatized risk behaviours delaying health care seeking and poorly developed health services [29-31]. About 50% - 80% of households in some communities of Far-western region have at least one family member working in India, and most of these migrants (mainly male) especially from Doti, Achham, Kailali, and Kanchanpur districts seasonally return home [32]. These migrants are usually the economic pillars of their families and most of the time of the year they stay outside of their homes, mainly in India for work [29]. Nevertheless, stigma and discrimination, risky behaviours (alcohol drinking), economic responsibility, guilt about going to brothels and acquiring HIV-infection when working abroad, and masculine thought of being strong and healthy might hinder them to come forward for early testing and treatment.

The association between low baseline bodyweight and mortality shown in this study is similar to several other studies, where low bodyweight or body mass index (BMI) was significantly associated with higher mortality [4,5,21,24]. Lower bodyweight is a proxy indicator of advanced disease (low CD4 and worst clinical stage) and risk factor of opportunistic infections like TB [24,33]. Malnutrition, poor immunity, and poor living standards, which are associated with low bodyweight, could also be responsible for the increased risk of mortality.

Adult HIV-infected patients who were bedridden for <50% or >50% of the days during the past month had higher risk of mortality compared to the patients with normal baseline performance status at treatment initiation. A study in Ethiopia showed similar findings, where bedridden performance status (not able to perform activities of daily living) was significantly associated with mortality [34]. Another study found no significant association, but when lost to follow-up patients were counted as death cases significant association was found [35]. Patients with advanced clinical diseases (WHO stage III or IV) had higher mortality compared to patients with WHO stage I or II. This finding was supported by several other studies done in Cameroon [4], Zambia [5], Ethiopia [20], Tanzania [24], and in other low-income countries [36]. The bedridden performance scale and clinical stage III or IV at ART initiation reflects the worst health condition of patients. Therefore, the significant effects of these conditions of patients on mortality indicate that patients died mostly because of their late initiation of ART when they had the worst health conditions.

The baseline CD4 count was not significantly associated with mortality in this study and this finding is consistent with one previous study [20]. However, most of the previous studies had contrast findings, where mortality varied significantly with baseline line CD4 count among HIV-infected patients. A study in India [6] showed that patients with CD4 count ≤50 had about three times higher mortality compared to >50 CD4 count. Another study in Tanzania [24] found higher mortality among patients with CD4 count <50 or 50–199 compared to >200 CD4 count. One more study in low-income countries found that HIV-infected patients with CD4 count less than 25 died about three times more than patients with ≥50 CD4 count [36]. In the present study most of the patients (86.4%) had CD4 count of >50 cells/mm^3^, which could have made the comparison with higher CD4 count statistically unstable. However, examining CD4 count as continuous variable did not show a significant association. Adjustment for the clinical stage and performance scale might have affected the association of CD4 count with mortality in multivariate analysis. Similarly, the adjustment for sex, and clinical characteristics (clinical stage, CD4 count, performance scale, and bodyweight) might have affected the independent effects of other variables (place of ART, age, active TB, ART regimen) which had significant effects on mortality in univariate analysis. Nonetheless, the variations in mortality rates across different ART sites, particularly the lower rates in Seti Zonal Hospital, could be attributed to the high rate (22.5%) of transfer-out at this site.

The main strength of this study is that it covers whole region. This study depicts the ART program outcome of diverse cultural and ecological regions (hill-difficult topography and plain/terai-easy access to health facility) of Nepal. This study could be applicable to other region within or outside Nepal with similar cultural and ecological context. The follow-up time was long enough to estimate survival and it determinants. This study used the routine treatment program data, which is cost effective and the findings would probably give a crucial insight to develop an effective and efficient HIV treatment, care, and support program and carved a sustainable way to respond HIV epidemic in Nepal. A limitation of this study is the lack of specific information on the causes of death. Another limitation is the lack of adequate information about Cotrimoxazole prophylaxis. Although it is routinely provided in Nepal, we were unable to include use of Cotrimoxazole prophylaxis in the analysis due to its incomplete information in the ART registers. Moreover, the mortality outcome of loss to follow-up patients in this study was unknown. Lost to follow-up patients might be at higher risk of death as shown in a meta-analysis performed in resource-limited settings that on average 46% of traced loss to follow-up patients on ART died [37]. However, a worst-case-scenario analysis was performed, where the mortality rate over the study period was estimated at 7.1 per 100 person-years at risk and the same determinants of mortality kept their significant association with increased mortality.

## Conclusions

Over the study period, mortality rate among HIV-infected patients was high. Majority of deaths were observed within 3 months of ART initiation. Higher mortality among adult HIV-infected patients was associated with male sex and poor baseline clinical characteristics i.e. bedridden baseline performance status, lower baseline bodyweight, advanced baseline clinical disease (WHO clinical stage III or IV). Patients (mainly male labour migrants in Far-western region) should be encouraged to come forward for early HIV testing and counseling, and to initiate early treatment. HIV-infected patients should to be given proper counseling on nutritional feeding.

## Competing interests

The authors declare that they have no competing interests.

## Authors’ contributions

LB, EK & LAA conceived and designed the study. LB, KD, RS & DKK participated in acquisition of data. LB analysed the data. LB, EK, KD, RS, DKK, AME & LAA interpreted the data and drafted the manuscript. All authors critically revised the manuscript and approved the final version. LB is the guarantor and takes responsibility for the integrity of the work as a whole.

## Pre-publication history

The pre-publication history for this paper can be accessed here:

http://www.biomedcentral.com/1471-2334/13/604/prepub
